# A robust stacked neural network approach for early and accurate breast cancer diagnosis

**DOI:** 10.3389/fmed.2025.1644857

**Published:** 2025-10-16

**Authors:** Xinkang Li, Menglong Gao, Chengyang Zhang, Guikai Ma, Qingyun Zhang, Wenjuan Meng, Tianbai Yuan, Yang Wang, Zhenhua Li

**Affiliations:** ^1^Department of Oncology, WeiFang People’s Hospital, Shandong Second Medical University, Weifang, Shandong, China; ^2^University of Colorado Denver, Denver, CO, United States; ^3^Department of Thyroid and Breast Surgery, WeiFang People’s Hospital, Shandong Second Medical University, Weifang, Shandong, China; ^4^Shanghai Clinical Research and Trial Center, ShanghaiTech University, Shanghai, China

**Keywords:** breast cancer, stacking ensemble, artificial neural network, classification, SHAP, clinical decision support

## Abstract

Timely and accurate diagnosis of breast cancer remains a critical clinical challenge. In this study, we propose Stacked Artificial Neural Network (StackANN), a robust stacking ensemble framework that integrates six classical machine learning classifiers with an Artificial Neural Network (ANN) meta-learner to enhance diagnostic precision and generalization. By incorporating the Synthetic Minority Over-Sampling Technique (SMOTE) to address class imbalance and employing SHapley Additive exPlanations (SHAP) for model interpretability. StackANN was comprehensively evaluated on Wisconsin Diagnostic Breast Cancer (WDBC) datasets, Ljubljana Breast Cancer (LBC) datasets and Wisconsin Breast Cancer Dataset (WBCD), as well as the METABRIC2 dataset for multi-subtype classification. Experimental results demonstrate that StackANN consistently outperforms individual classifiers and existing hybrid models, achieving near-perfect Recall and Area Under the Curve (AUC) values while maintaining balanced overall performance. Importantly, feature attribution analysis confirmed strong alignment with clinical diagnostic criteria, emphasizing tumor malignancy, size, and morphology as key determinants. These findings highlight StackANN as a reliable, interpretable, and clinically relevant tool with significant potential for early screening, subtype classification, and personalized treatment planning in breast cancer care.

## 1 Introduction

Cancer is a major disease that seriously threatens human health worldwide, and breast cancer is particularly common among women (1). Breast cancer is the most common cancer in the world. Breast cancer is the most common type of cancer in the world, with more than 2.3 million new cases diagnosed in 2020 and approximately 685,000 deaths (2). Since the early symptoms of breast cancer are relatively hidden, many patients do not feel obvious discomfort in the early stage, and the disease is often discovered in the late stage, resulting in missing the best treatment opportunity. Therefore, early diagnosis of breast cancer is very important, which is directly related to the patient’s survival rate and cure rate (3).

Traditional breast cancer diagnosis methods, such as CT, mammography, magnetic resonance imaging (MRI), ultrasound, and Fine Needle Aspiration (FNA), are widely used in clinical practice (4). However, these methods heavily rely on the doctor’s experience and judgment, which are influenced by subjective factors (5). This is particularly problematic when the tumor boundary is unclear, or the lesion is in its early stages, where misdiagnosis or missed diagnosis can occur. Furthermore, long working hours and fatigue may lead to increased analysis errors. Despite their broad application, these methods face significant challenges in accuracy and reliability, particularly in complex cases or when early-stage detection is critical. This sets the stage for exploring more robust and objective diagnostic approaches, such as machine learning-based models (6). Nowadays, with the rapid development of machine learning technology, the field of breast cancer diagnosis has ushered in new breakthroughs. Machine learning can automatically extract hidden patterns and features through deep learning of large amounts of clinical data and imaging data, thereby achieving more accurate breast cancer prediction and classification (7). Compared with traditional diagnostic methods that rely on expert experience, machine learning improves the stability and consistency of diagnosis, reduces the impact of human factors, and significantly reduces the risk of misdiagnosis and missed diagnosis by doctors (8). Combining traditional methods with machine learning technology can improve diagnostic efficiency, help doctors make more objective and accurate judgments, and promote the realization of early diagnosis and personalized treatment.

In recent years, ensemble learning methods have evolved from traditional strategies such as Bagging and Boosting to more complex and efficient fusion models (9). Among these, the stacking method, also known as stacking generalization, has emerged as a popular research approach. However, many stacking models still rely on relatively simple algorithms and do not fully exploit the potential of multi-model fusion. While stacking methods improve the model’s ability to handle data features by integrating various algorithms [e.g., the linear discriminant of Logistic Regression (LR), the anti-interference ability of Random Forest (RF), and the boundary demarcation ability of Support Vector Machine (SVM)], they often struggle to address issues like class imbalance and high-dimensional data interactions (10). Moreover, these models may fail to provide a robust solution in real-world clinical settings where the data is often noisy and imbalanced. At present, some breast cancer classification studies have integrated features, but they have not adopted the stacking method. Among them, the hybrid ensemble model has become a development trend (11, 12). For example, hybrid models such as the hybrid of traditional machine learning models and deep learning models, and the hybrid of traditional methods and machine learning, can improve the high-dimensional fusion and learning capabilities of data. This hybrid strategy can achieve more efficient and accurate predictions in practical applications while taking into account the advantages of different models. For example, the method proposed by Murat Karabatak et al. combines association rules and neural networks, which is a hybrid integration method of feature selection and classifier (13). The FS-WOA-Stacking model proposed by Shanshan Kong et al. integrates five mainstream machine learning models: SVM, ANN, RF, eXtreme Gradient Boosting (XGBoost) and Adaptive Boosting (AdaBoost), and combines feature selection and whale optimization algorithm (WOA) optimization for early diagnosis of breast cancer (14). The hybrid integration method has certain advantages in improving model performance, but it still lags behind the stacking method in the deep application of multi-model fusion and meta-learning strategies. However, machine learning methods have been widely applied in breast cancer diagnosis, many of these studies still rely on traditional models [such as SVM and K-Nearest Neighbors (KNN)] and identical datasets (e.g., WDBC and LBC), which limits their ability to generalize across different clinical settings and improve accuracy in complex cases (9, 15). Traditional single algorithms struggle to capture complex data patterns and feature interactions, often leading to overfitting or poor generalization (15). Furthermore, existing hybrid models often fail to fully leverage multi-model fusion techniques or address critical issues like class imbalance and the high-dimensional nature of medical datasets. In existing studies, Maldonado et al. (16) proposed REF-SVM, which simultaneously performs feature selection and classification using kernel-penalized support vector machines. While effective in reducing feature dimensionality, this model remains sensitive to class imbalance and relies heavily on kernel function selection. Kumar and Poonkodi (17) attempted a hybrid RF + KNN + SVM model, which simply combines multiple classifiers but lacks a meta-learning mechanism, making it difficult to optimize feature interactions and address class imbalance. Idri et al. (18) investigated Uniform Multilayer Perceptron (UMLP) and Evolutionary Parameter-tuned Multilayer Perceptron (EPMLP). Although these methods improve performance through structural adjustments and parameter evolution, they suffer from limited interpretability and high computational costs. None of these approaches systematically address the synergistic challenges of high-dimensional feature interactions, class imbalance, and clinical interpretability in breast cancer diagnosis. To address these gaps, we propose StackANN, a multi-model stacking approach integrated with the SMOTE. This method combines multiple classical machine learning models and employs an ANN as a meta-learner. By effectively handling complex data patterns, feature interactions, and class imbalance, StackANN aims to improve classification accuracy and demonstrates stronger robustness and generalization ability compared to existing approaches.

## 2 Methods

### 2.1 Datasets

The LBC Dataset was provided by the Institute of Oncology, University Medical Center, Ljubljana, Yugoslavia (19). This dataset contains clinical sample information from 286 breast cancer patients. Each sample contains 9 clinical features related to breast cancer prognosis (such as tumor size, lymph node capsule, etc.) and a label that identifies the sample category (0 for benign and 1 for malignant). Among them, there are 201 benign samples and 85 malignant recurrence samples. In this paper, we performed necessary data preprocessing on the LBC Dataset. The detailed process of data preprocessing is shown in the supporting materials.

The WDBC Dataset comes from the UCI Machine Learning Library and contains 569 breast cancer samples, including 212 benign samples and 357 malignant samples (20). Each sample consists of 30 features and 1 label, with label value B indicating benign (0) and label value M indicating malignant (1). The features are obtained through FNA and mainly describe the morphological characteristics of tumor cell nuclei, including area, smoothness, and texture.

Prior to machine learning modeling, we standardized both datasets using Z-score normalization to ensure all features were on a comparable scale. This preprocessing step centers the data to zero mean and unit variance, facilitating stable and efficient model convergence. The standardized data was used for all subsequent training and evaluation processes of both base learners and the meta-learner in our stacking ensemble framework. The datasets were randomly divided into training and test sets at a ratio of 80 and 20% (Table 1) for training and performance evaluation of the baseline model. The training set was used to train the model, and the test set was used to evaluate the performance of the final training model on unknown data.

**Table 1:** Partitioning of LBC and WDBC datasets.

Datasets	Category	Malignant	Benign	Total number
LBC	Training	64	164	228
	Test	21	37	58
WDBC	Training	286	169	455
	Test	71	43	114

In order to verify the generalization performance of the model, we selected the WBCD dataset. The WBCD dataset is a dataset commonly used in breast cancer classification research (21). It contains 699 breast cancer samples from the University of Wisconsin Medical Center, of which 458 samples are benign (0) and 241 samples are malignant (1). Each sample consists of 9 features and 1 label. The features are obtained through fine FNA and mainly describe the morphological characteristics of tumor cell nuclei, including radius, texture, smoothness, perimeter, area, etc. To ensure data quality, samples containing missing values were removed, and the final dataset contains 683 valid samples. We use this dataset as an external validation set.

### 2.2 Machine learning model

This paper studies various machine learning models for binary classification tasks (malignant and benign). KNN calculates the distance between samples, selects the K nearest neighbors, and uses a majority voting mechanism to determine the sample category (22, 23). SVM is a supervised learning method that achieves optimal classification of sample data by constructing a hyperplane that maximizes the interval between different categories (24). AdaBoost is an enhancement algorithm that repeatedly trains multiple weak classifiers and adjusts sample weights in each iteration to increase attention to misclassified samples, thereby effectively improving the overall classification performance of the model (25, 26). RF reduces overfitting and improves prediction stability by constructing multiple decision trees and outputting the final results by voting or averaging (27). XGBoost is an ensemble learning algorithm based on gradient boosting. It iteratively builds decision trees and corrects the previous round of prediction errors, while combining regularization techniques to improve the accuracy and generalization ability of the model (28, 29). DT achieves classification by recursively splitting the feature space and selecting the optimal split point according to the sample characteristics (30).

This article uses these six machine learning models as baseline models and applies them to two datasets. The entire computational process uses the Python 3.11 environment, and the scikit-learn library is used to implement the training and evaluation of machine learning models. To ensure a fair comparison of model performance, all base learners were optimized using the same hyperparameters. The detailed optimal configurations for both datasets are shown in Supplementary Table S1 in the supporting materials.

### 2.3 Stacked ensemble method

The stacking method is a special ensemble learning method (31). The performance of a single model under different data distributions may be unstable, and the stacking method can effectively make up for the limitations of a single model by integrating the prediction results of multiple models (32).

The StackANN constructed in this paper has two layers, the first layer is the base learner, and the second layer is the meta learner. In the first layer, multiple base models are trained on the training data at the same time. Each base learner generates corresponding prediction results based on the training data. These prediction results have two forms: category labels or category probabilities. These prediction results have two forms: category labels or category probabilities. We choose category probabilities as the prediction results because they provide richer information about the model’s confidence, which can help the stacking ANN better integrate base learner outputs and improve overall predictive performance. In the second layer, the prediction results of the six base learners are used as six new features, and the corresponding true labels are used as target features to form a new dataset. However, we found that the generated new dataset has a serious class imbalance problem, which may cause the model to over-rely on majority class samples and perform poorly on minority classes. Therefore, to address this issue and ensure methodological rigor, we implemented the following processing pipeline before training the base learners:

1. Applied the SMOTE (33) to the original dataset to generate a balanced dataset;
2. Split the balanced dataset into training and testing sets;
3. Trained all base learners using the training set and generated prediction probabilities on the testing set;
4. Combined the prediction probabilities from each base learner on the testing set with the true labels to construct a meta-dataset;
5. Performed an additional split on the meta-dataset and employed an ANN as the meta-learner for final training and prediction.

This approach effectively balances the distribution between benign and malignant cases while strictly preventing information leakage between training and testing phases (34). The consistent use of optimized splitting ratios ensures coherence across different model levels.

Compared to a single model, StackANN requires training multiple base learners and one meta-learner, thus incurring higher computational and time costs during the offline training phase. However, this cost is justified by a significant improvement in classification performance—particularly in Recall, a critical clinical metric for reducing missed diagnoses. During the online prediction stage after deployment, the computational overhead remains comparable to that of a conventional single model, ensuring no practical impact on the efficiency of real-time clinical applications.

### 2.4 ANN as meta-learner

ANN is a computational model that simulates the biological nervous system. It processes data through multiple interconnected neuron hierarchies, imitating the information transmission and processing methods of biological neurons (35). In the second layer of the StackANN model, ANN is used as a meta-learner to integrate the prediction results of the base learners and make the final classification decision. We use an ANN consisting of an input layer, multiple hidden layers, and an output layer. The input layer contains 6 nodes, corresponding to the prediction results of the 6 base learners in the first layer. The LBC dataset uses 4 hidden layers, while the WDBC dataset uses 3 hidden layers. Each hidden layer has several neurons and uses the ReLU activation function, as shown in Equation 1. Its function is to set the part of the input less than 0 to 0 and keep the part of the input greater than 0 unchanged, thereby enhancing the nonlinear expression ability of the model (36).

(1) ReLU(x)=max(0,x)

The output layer uses the Softmax activation function to convert the output of the model into a probability distribution, that is, the sum of the predicted probabilities of each category is 1. In the binary classification task, the category with a higher probability is selected as the final prediction result of the model, thereby achieving classification judgment of the sample. As shown in Equation 2, where $z_{i}$ denotes the input score of class i , and K is the total number of classes.

(2) $$\text{Softmax}(z_{i})=\frac{e^{z_{i}}}{\sum_{j=1}^{K}e^{z_{j}}},\;\;\text{for}\;i=1,2,\ldots ,K$$

During the model training process, Binary Cross-Entropy Loss (also known as Log Loss) is selected as the loss function for supervised training to measure the difference between the model’s predicted probability and the true binary classification label. As shown in Equation 3, where N is the number of samples, $y_{i}$ is the true label of sample i , which can be either 0 or 1, and $p_{i}$ is the predicted probability that sample i belongs to the positive class.

(3) $$\text{Loss}=-\frac{1}{N}\sum_{i=1}^{N}(y_{i}\cdot \log(p_{i})+(1-y_{i})\cdot \log(1-p_{i}))$$

The optimizer used is Adaptive Moment Estimation (Adam) method, which combines Momentum and Root Mean Square Propagation (RMSProp) algorithms to improve training efficiency and accelerate model convergence. The update formula of Adam is as follows (see Equation 4).

(4) $$\theta_{t}=\theta_{t-1}-\frac{\eta }{\sqrt{{\hat{v}}_{t}}+\epsilon }{\hat{m}}_{t}$$

In which, $\theta_{t}$ is the parameter at the current moment, η is the learning rate, ${\hat{m}}_{t}$ is the first moment estimate of the gradient (i.e., the momentum term), ${\hat{v}}_{t}$ is the second moment estimate of the gradient (i.e., the weighted variance term), and ϵ is a small constant that prevents division by zero.

**Figure 1:**
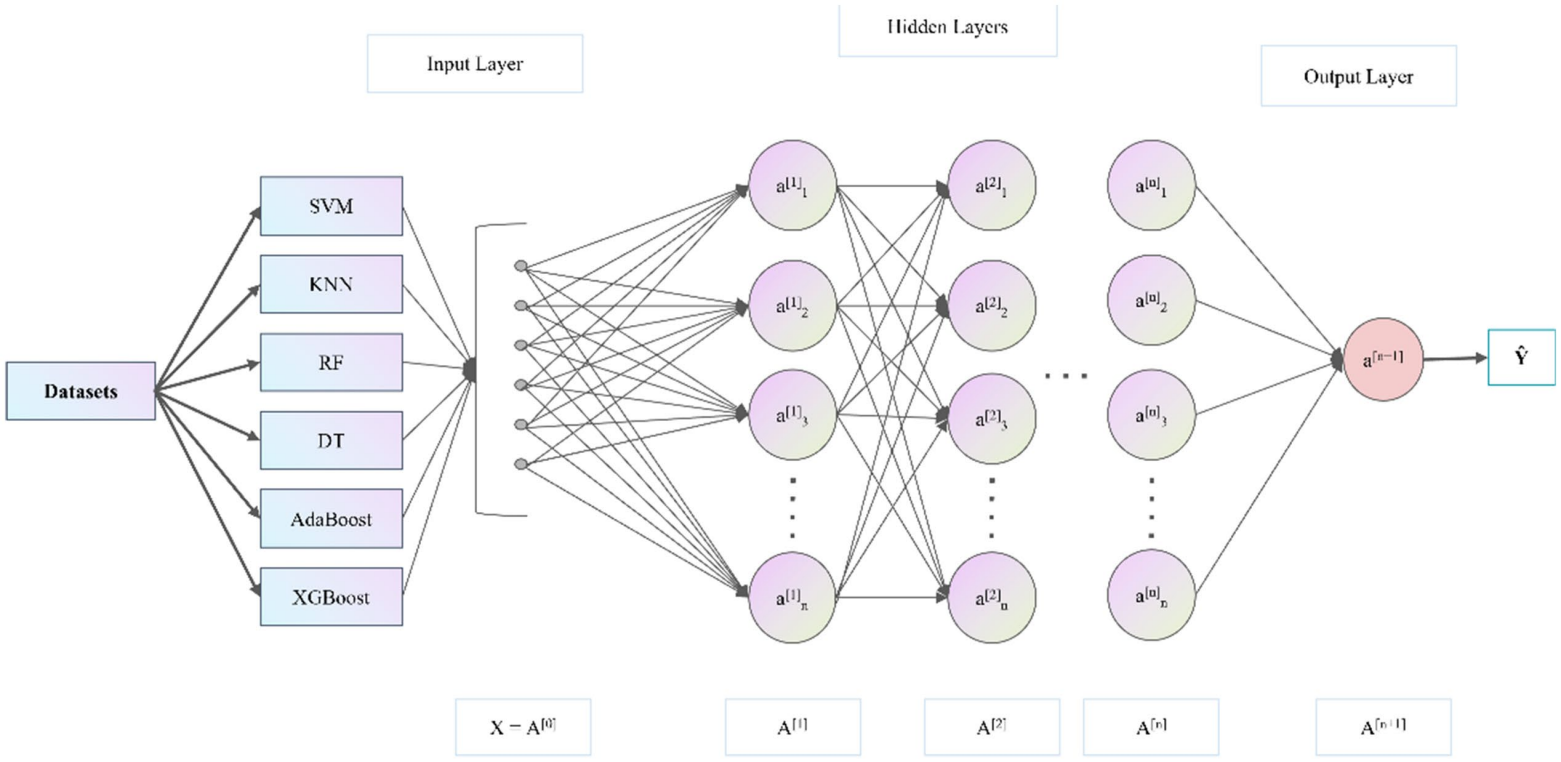
The design of StackANN.

All training processes were carried out in Python 3.11. The specific method framework is shown in Figure 1, which shows the hierarchical structure and information transmission process of the StackANN model. In order to optimize the model performance, we optimized the hidden layer structure, learning rate, maximum number of iterations and other hyperparameters during the setting process. The detailed hyperparameters are listed in Table 2. Here, Hidden Layer Sizes refers to the number of neurons in each hidden layer, reflecting the network architecture; Max Iterations is the maximum number of weight update iterations during training; Alpha is the L2 regularization parameter, used to prevent overfitting; Learning Rate Init represents the initial step size of the learning rate at the start of training. To ensure the reproducibility of experimental results, we fixed the random seed throughout the entire experimental workflow: the global random seed was set to 3,407 to control all major random processes (including SMOTE oversampling and model initialization); the data splitting process random seed was set to 42 to ensure consistent training-test splits. This two-level seeding strategy guarantees complete reproducibility of results while adhering to best practices in experimental design.

**Table 2:** Hyperparameter table for ANN.

Models.Method	Hidden layer sizes	Max iterations	Alpha	Learning rate
LBC	(200,150,100,50)	400	0.0001	0.001
WDBC	(100,50,25)	300	1.0000	0.001

To address these gaps in existing methods, we propose StackANN, a novel classification method based on multi-model ensemble learning. While previous studies have relied on traditional classifiers and basic ensemble strategies, StackANN integrates six classical machine learning models [KNN, AdaBoost, SVM, RF, XGBoost, and Decision Tree (DT)] and uses an ANN as a meta-learner. This approach enhances classification performance by leveraging the complementary strengths of various base models and improving generalization ability, particularly in complex, high-dimensional, and imbalanced datasets. Unlike existing hybrid models, which fail to fully address class imbalance or complex feature interactions, StackANN captures higher-order feature relationships through the meta-learning process with ANN, optimizing the decision boundary via nonlinear transformation. To demonstrate the effectiveness of StackANN, we conducted experiments on the LBC and WDBC datasets, and also performed external validation on the WBCD datasets. The results demonstrate that StackANN significantly outperforms single models in classification accuracy and robustness. Furthermore, on the external validation set (WBCD), StackANN achieved excellent performance and demonstrated good generalization. This result further confirms that StackANN provides an efficient and robust solution for complex classification tasks, outperforming existing hybrid models in handling data complexity, class imbalance, and feature interactions. Our findings highlight the potential of StackANN as a clinically applicable, interpretable, and generalizable model for breast cancer diagnosis.

### 2.5 Evaluation metrics

In order to better evaluate the model performance and stability of the two datasets, this study used several common evaluation indicators: Accuracy (ACC) (37), Precision (Pre) (38), Recall (39), F1-score (F1) (40), Specificity (Sp) (41) and AUC (42), these indicators can reflect the performance of the model in classification tasks from different angles. We define four basic classification results: True Positive (TP), True Negative (TN), False Positive (FP) and False Negative (FN). These four values constitute the Confusion Matrix, which provides the basis for various evaluation indicators (43). Specifically, we use the same evaluation indicators to evaluate the sample classification results of the two datasets and compare them with the original processing results. The specific evaluation indicators are as follows:

ACC is a common indicator for evaluating the overall performance of a model, indicating the proportion of correctly classified samples to the total number of samples. The value ranges from 0 to 1, and the closer it is to 1, the better the model performs in the classification task. The calculation formula is as shown in Equation 5.

(5) $$\mathrm{ACC}=\frac{\mathrm{TP}+\mathrm{TN}}{\mathrm{TP}+\mathrm{TN}+\mathrm{FP}+\mathrm{FN}}$$

Pre measures the proportion of samples that are actually positive among those predicted by the model to be positive. The higher the value, the more accurate the model is in predicting positive classes. The calculation formula is as shown in Equation 6.

(6) $$\mathrm{Pre}=\frac{\mathrm{TP}}{\mathrm{TP}+\mathrm{FP}}$$

Recall measures the proportion of samples that are actually positive that are successfully classified as positive by the model. The higher the value, the stronger the model is in identifying positive samples. The calculation formula is as shown in Equation 7.

(7) $$\text{Recall}=\frac{\mathrm{TP}}{\mathrm{TP}+\mathrm{FN}}$$

F1 is the harmonic mean of Precision and Recall, which aims to measure the balance between the two. If one of the indicators is low, F1 will also decrease accordingly, thus avoiding the situation where the model is biased toward one category. The calculation formula is as shown in Equation 8.

(8) $$F1=\frac{2\mathrm{TP}}{2\mathrm{TP}+\mathrm{FP}+\mathrm{FN}}$$

Sp reflects the proportion of samples that are correctly predicted as negative among all samples that are actually negative. The higher the Sp, the fewer FPs, and the better the model performs on negative samples. The calculation formula is as shown in Equation 9.

(9) $$\mathrm{Sp}=\frac{\mathrm{TN}}{\mathrm{TN}+\mathrm{FP}}$$

The Receiver Operating Characteristic (ROC) curve is a curve drawn with the False Positive Rate (FPR) (see Equation 10) as the horizontal axis and the True Positive Rate (TPR, i.e., Recall) as the vertical axis. The closer the ROC curve is to the upper left corner (i.e., high TPR and low FPR), the better the model performance.

(10) $$\mathrm{FPR}=\frac{\mathrm{FP}}{\mathrm{FP}+\mathrm{TN}}$$

AUC represents the area under the ROC curve. The calculation of the area is shown in Equation 11. AUC is a key indicator for measuring the performance of a binary classification model, which comprehensively reflects the performance of the model under different classification thresholds. Its value range is between 0 and 1. The closer the value is to 1, the better the classification performance of the model is, and it has a stronger ability to distinguish between positive and negative samples. Specifically, when the value is 1, the model can perfectly distinguish between positive and negative samples under all thresholds, while when the value is 0.5, it means that the performance of the model is equivalent to random guessing and lacks effective discrimination ability.

(11) $$\mathrm{AUC}=\frac{1}{2}\sum_{i=1}^{n-1}({\mathrm{TPR}}_{i}+\mathrm{TP}R_{i+1})\times (\mathrm{FP}R_{i+1}-{\mathrm{FPR}}_{i})$$

## 3 Results and discussion

### 3.1 Model performance analysis

**Figure 2:**
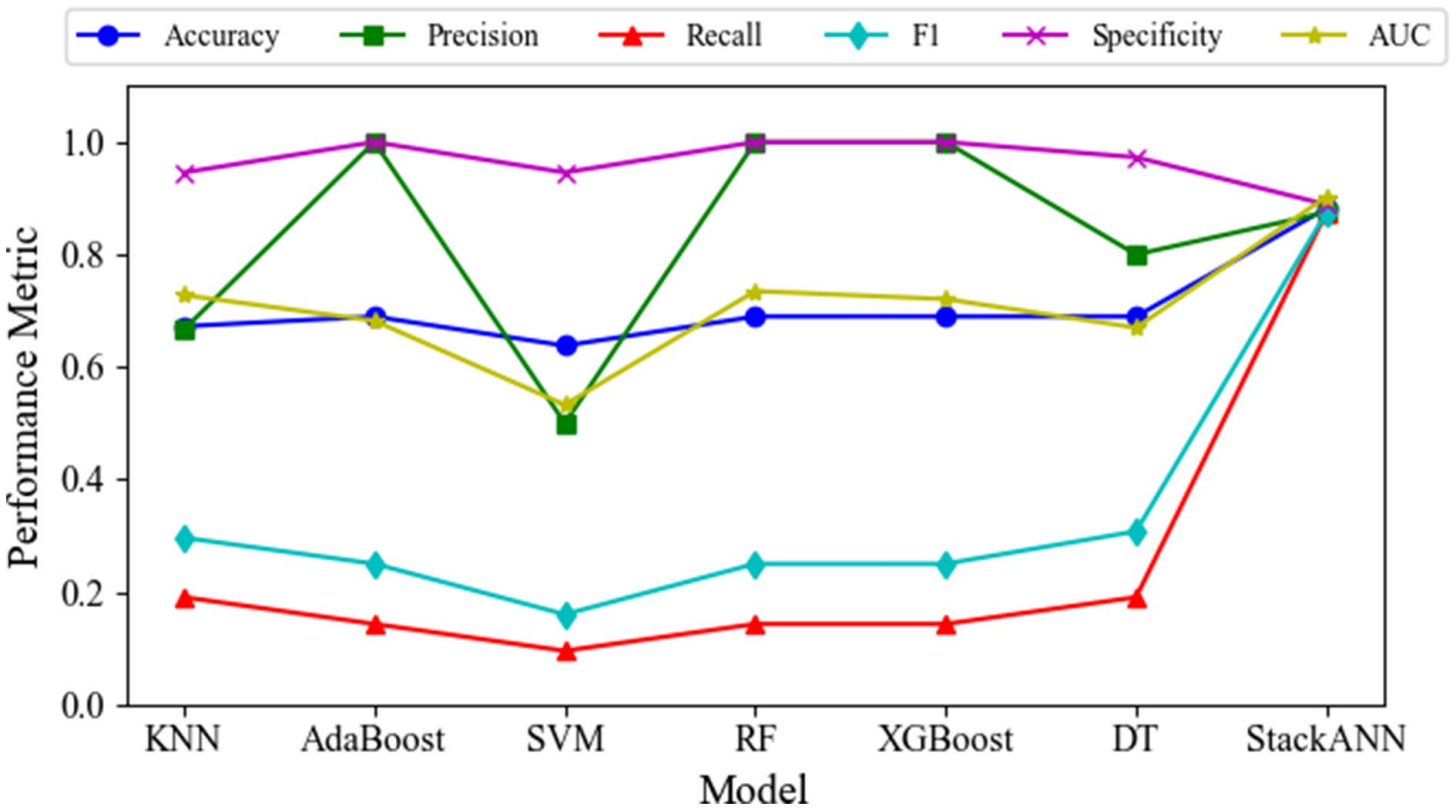
Variation of multiple evaluation metrics for different models on the LBC dataset.

To verify the effectiveness of the model, this paper systematically compares and analyzes the proposed StackANN, six typical machine learning baseline models and existing research methods from multiple performance dimensions based on the LBC and WDBC datasets. For the specific evaluation indicators of each model on the LBC dataset, see Table 3. The ACC of the StackANN model reached 0.8824, and the AUC value was 0.9028, both of which were better than all the baseline models compared, indicating that the model showed stronger advantages in overall classification performance and the ability to distinguish between positive and negative samples. The Pre value of the model was 0.8750, which was higher than that of KNN, SVM and DT, but lower than that of AdaBoost, XGBooost and RF (1.0000), indicating that the ACC of the model in predicting malignant tumors was at a medium level compared with the baseline model, and there was a certain degree of false positives (slightly lower Sp value). However, its Recall and F1 are better than the baseline model, that is, the comprehensive ability of the model to identify malignant tumors is stronger than that of the baseline model. In particular, Recall has been significantly improved (see the broken line change of the Recall indicator in Figure 2). The Recall of the baseline model is lower than 0.2000, while the Recall of the StackANN model is as high as 0.8750. In addition, the performance of the various indicators of the StackANN model is relatively balanced. Compared with the baseline model, the StackANN model has better capabilities in all aspects and does not overly ignore the optimization of other indicators. The performance change trends of different indicators of each model are shown in Figure 2. Further analysis shows that StackANN may focus more on the improvement of Recall during the training optimization process of the LBC dataset, that is, by accepting some false positives in exchange for higher positive Recall capabilities. Although Pre has not been improved, the overall performance of the model in positive recognition has been enhanced, showing stronger practicality and robustness. In medical scenarios, high Recall means that the model can identify most real malignant tumor samples. Even if some benign tumors are misclassified as malignant (false positives), it can avoid missed diagnoses to the greatest extent and has important clinical value.

**Table 3:** Performance comparison of breast cancer classification models on the LBC dataset.

Method	ACC	Pre	Recall	F1	Sp	AUC
KNN	0.6724	0.6667	0.1905	0.2963	0.9459	0.7278
AdaBoost	0.6897	1.0000	0.1429	0.2500	1.0000	0.6821
SVM	0.6379	0.5000	0.0952	0.1600	0.9459	0.5328
RF	0.6897	1.0000	0.1429	0.2500	1.0000	0.7349
XGBoost	0.6897	1.0000	0.1429	0.2500	1.0000	0.7207
DT	0.6897	0.8000	0.1905	0.3077	0.9730	0.6699
StackANN	0.8824	0.8750	0.8750	0.8750	0.8889	0.9028

**Figure 3:**
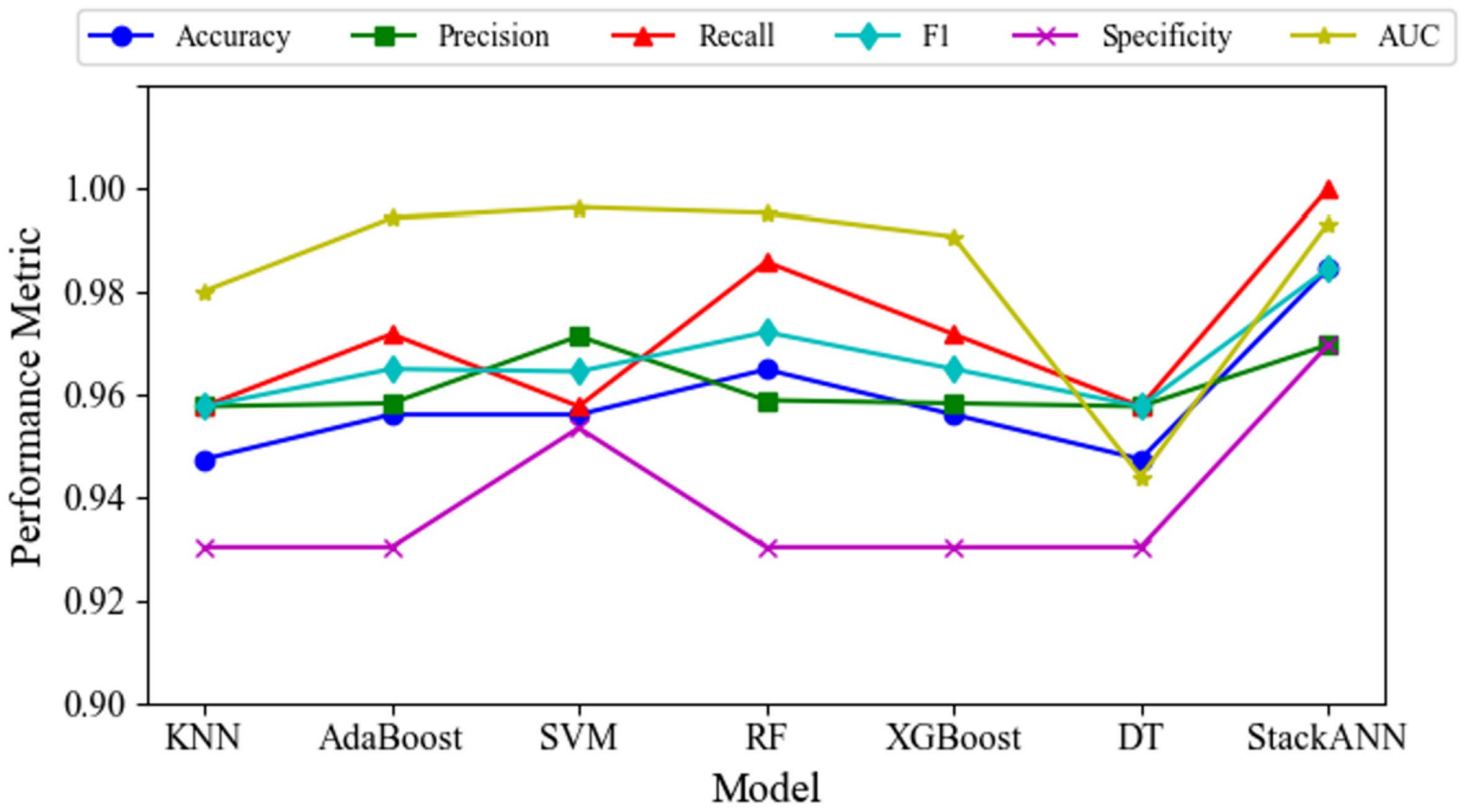
Variation of multiple evaluation metrics for different models on the WDBC dataset.

Experimental results on the WDBC dataset demonstrate that the StackANN model exhibits significant advantages across multiple key classification metrics. The model achieves an ACC of 0.9847 and an AUC of 0.9934, reflecting its excellent overall classification performance and ability to distinguish between categories. Particularly noteworthy is its Recall of 1.0000, indicating that all malignant tumor samples were correctly identified with no missed diagnoses, significantly reducing medical risks. The Pre is 0.9697, showing that the vast majority of samples predicted as malignant are true positives. Similarly, the Sp is 0.9697, indicating high ACC in identifying benign tumors. The harmonic mean F1 score of Pre and Recall is 0.9847, further highlighting the model’s outstanding comprehensive performance in classifying positive samples. The performance change trends of different indicators of each model are shown in Figure 3.

Compared to traditional machine learning models, the StackANN ensemble model demonstrates comprehensive superiority. Both KNN and DT exhibit significantly lower Recall and F1 scores than StackANN. Although AdaBoost, XGBoost, and SVM perform similarly in terms of Pre, their Recall remains below 1.0000, indicating a risk of missed diagnoses. While Random Forest (RF) achieves a relatively high Recall (0.9859), its overall F1 score and Recall still fall short of StackANN. Compared with recently proposed hybrid and deep learning models, StackANN demonstrates superior overall performance in terms of ACC and F1. Specifically, StackANN achieves an ACC of 0.9846, significantly higher than UMLP (0.9578), and EPMLP (0.9701). In terms of F1, StackANN (0.9846) also outperforms UMLP (0.9580) and EPMLP (0.9705). Importantly, StackANN achieves a perfect Recall of 1.0000 while maintaining high ACC, indicating that the model can comprehensively identify all malignant samples, thereby substantially reducing the risk of missed diagnoses in clinical settings. In addition, the close alignment between its ACC and F1 indicates an optimal balance between Pre and Recall, a critical characteristic in medical diagnostic scenarios where both false positives and false negatives have significant clinical implications. These results fully demonstrate that StackANN possesses stronger generalization capability and stability. The specific evaluation indicators of each model are shown in Table 4.

**Table 4:** Performance comparison of breast cancer classification models on the WDBC dataset.

Method	ACC	Pre	Recall	F1	Sp	AUC
KNN	0.9474	0.9577	0.9577	0.9577	0.9302	0.9802
AdaBoost	0.9561	0.9583	0.9718	0.9650	0.9302	0.9944
SVM	0.9561	0.9714	0.9577	0.9645	0.9535	0.9964
RF	0.9649	0.9589	0.9859	0.9722	0.9302	0.9953
XGBoost	0.9561	0.9583	0.9718	0.9650	0.9302	0.9908
DT	0.9474	0.9577	0.9577	0.9577	0.9302	0.9440
UMLP (18)	0.9578	0.9580	0.9580	0.9580		
EPMLP (18)	0.9701	0.9710	0.9700	0.9705		
StackANN	0.9846	0.9697	1.0000	0.9846	0.9697	0.9934

### 3.2 SHAP-based multi-model feature attribution analysis for breast Cancer classification

To analyze the impact of features on the model’s prediction results, this study employs the SHAP method to interpret the feature importance of the StackANN model (44). Specifically, six baseline models are trained separately, and the KernelExplainer interpreter is used on the same test samples to calculate the SHAP value of each model. Then, the SHAP values output by all models are averaged element by element in the feature dimension to obtain the global average SHAP value of each feature, which is used as the basis for the comprehensive feature interpretation of the StackANN model, and a SHAP bee swarm diagram is drawn for visual analysis. In the bee swarm diagram, the X-axis represents the SHAP value of each feature, indicating the contribution of the feature to the prediction result. A single point represents the SHAP value of a sample on the feature. The Y-axis is the feature name, which is sorted from top to bottom by the absolute value of the average SHAP value (the more important, the higher the value). The color represents the original value of the feature, red means that the value of the feature is large, and blue means it is small. By fusing the interpretation results of multiple models, it helps to alleviate the bias that may be caused by the interpretation of a single model and improves the credibility of the importance of the feature.

**Figure 4:**
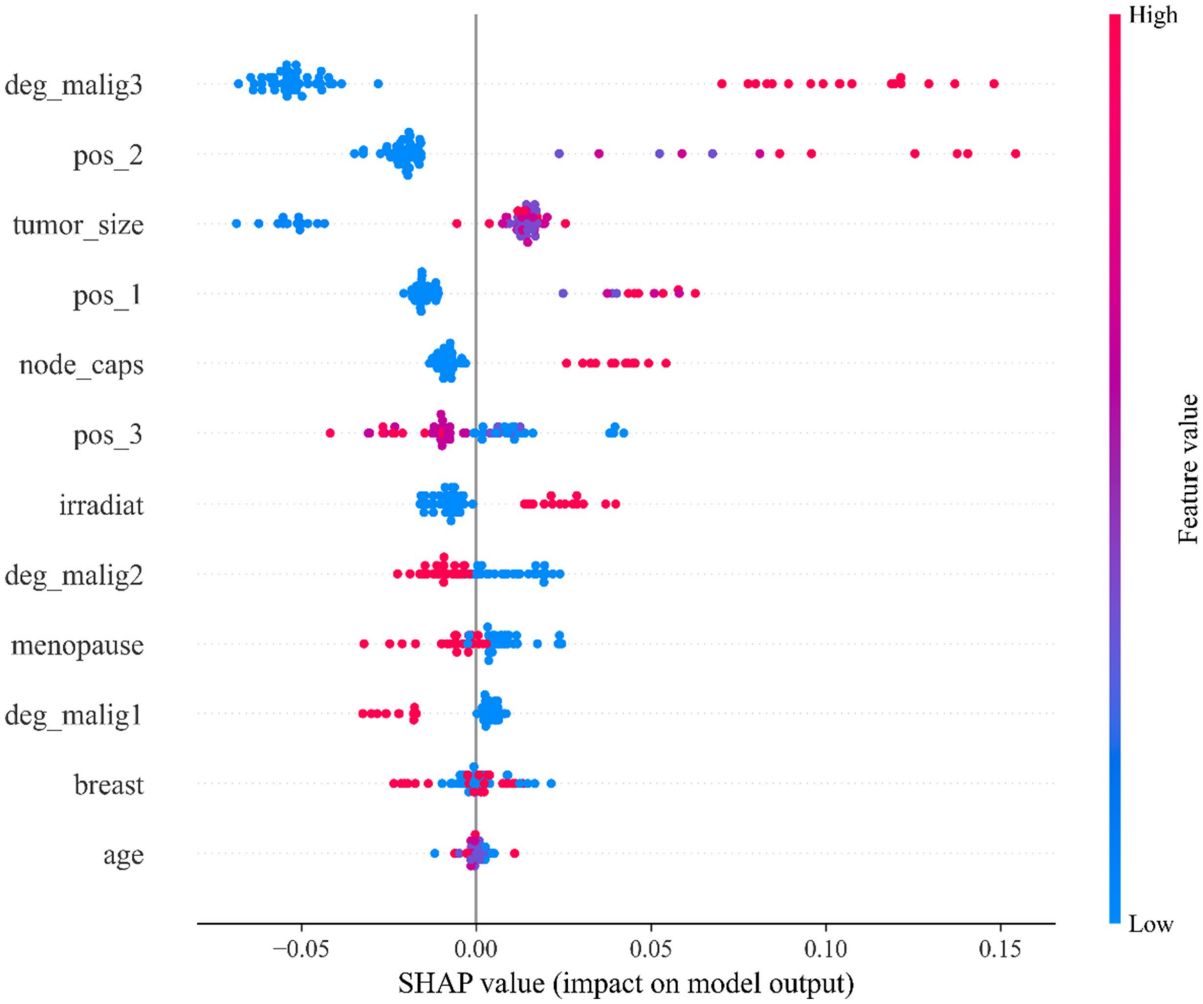
SHAP feature importance beeswarm plot of the StackANN model on the LBC dataset.

As can be observed from Figure 4, in the LBC dataset, the model mainly relies on clinical features such as tumor malignancy, location, and size for prediction. Specifically, feature deg_malig3 (malignancy level 3) is the feature with the greatest impact on the model output, followed by feature pos_2 (position 2) and feature tumor_size (tumor size), while demographic features such as age and breast location have relatively small impacts. The points of top features such as deg_malig3, pos_2, and tumor_size are widely distributed, indicating that they have significant effects on different samples to varying degrees. Feature deg_malig3 represents the highest level in histological grading. Grade 3 represents the most poorly differentiated and most malignant tumor, reflecting the high degree of atypia and poor differentiation of tumor cells. Clinically, it usually represents the most aggressive and worst prognostic pathological type (45). Therefore, it plays a decisive role in the prediction model. The feature pos_2 reveals the specific location of the tumor in the breast, which affects its prognosis and malignancy. The feature tumor_size is a key indicator to measure the growth potential of the tumor, which directly affects the malignancy and prediction results. In summary, the model mainly relies on the biological behavior characteristics of the tumor for prediction, especially key factors such as histological grade, tumor location and size.

**Figure 5:**
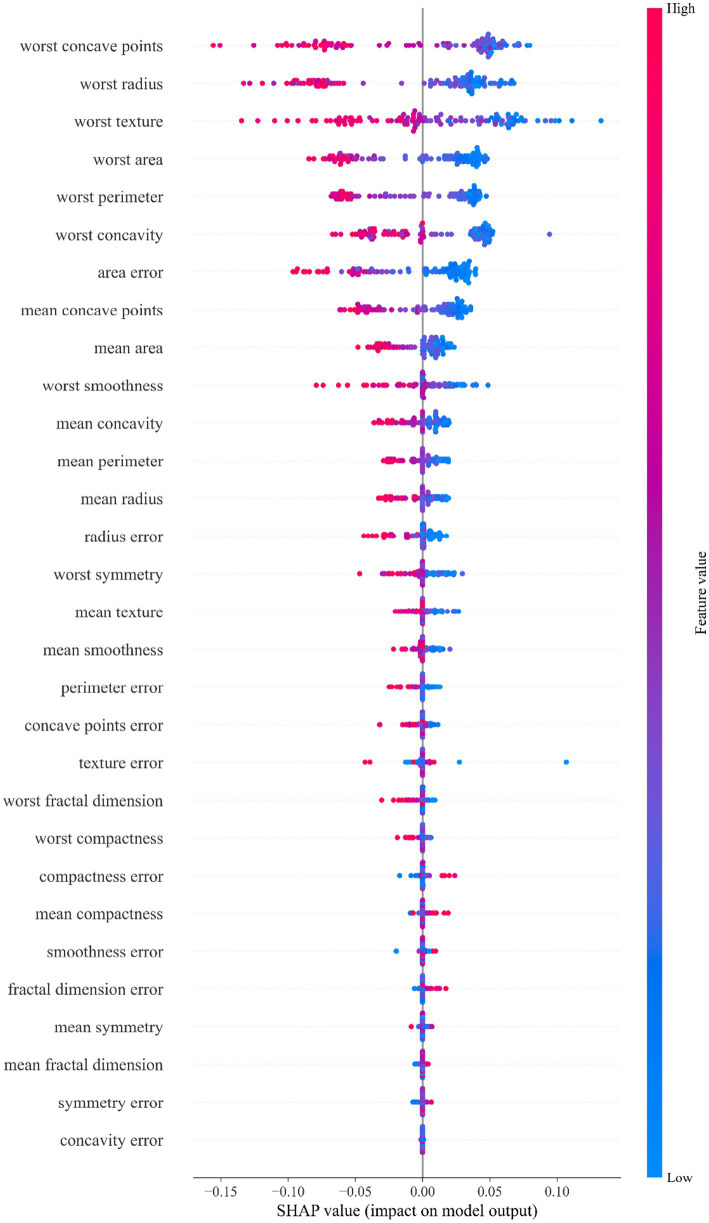
SHAP feature importance beeswarm plot for the StackANN model on the WDBC dataset.

The analysis results of Figure 5 show that in the WDBC dataset, the morphological features of the most severe tumor area play a dominant role in model prediction. Among them, the feature “worst concave points” was identified as the most influential predictor, with the widest distribution of SHAP values and the highest contribution. This feature reflects the degree of concavity of the tumor contour. More or deeper concavities usually mean irregular tumor boundaries, suggesting stronger invasive growth potential and higher risk of malignancy. The important features that follow closely include: “worst radius,” “worst texture” and “worst area.” Among them, the feature “worst radius” reflects the maximum size of the tumor and is closely related to the volume of the lesion; the feature “worst texture” measures the complexity of the texture of the tumor area, and uneven grayscale indicates enhanced tissue structural heterogeneity; and the feature “worst area” represents the maximum projection area of the lesion in the image, which can also be regarded as an intuitive indicator of the extension range of the tumor. It is worth noting that all features prefixed with “worst” (representing the most extreme state of the tumor) generally contribute more to the model than the average features prefixed with “mean,” indicating that the model relies more on identifying the most malignant areas of the tumor. This trend is highly consistent with the clinical diagnostic strategy of focusing on the most invasive and malignant areas (45). In addition, various error features (such as area error, etc.) contribute relatively little to model prediction, suggesting that the absolute level of features (such as maximum value) is more valuable for clinical judgment than its volatility (error). The above feature importance ranking provides a valuable reference for clinical practice and an important reference for intelligent diagnosis of breast cancer, indicating that in the actual judgment process, we should focus on indicators such as tumor edge morphology, size and structural heterogeneity.

### 3.3 Analysis of model classification effect

The confusion matrix is an important tool for evaluating the classification performance of a model (46), it visually shows how the model’s predictions for both positive and negative classes compare to the true labels. To more comprehensively analyze the classification effects of each model, we plotted confusion matrices for the LBC and WDBC datasets, respectively, to further reveal the recognition capabilities and classification biases of the models on different types of samples.

**Figure 6:**
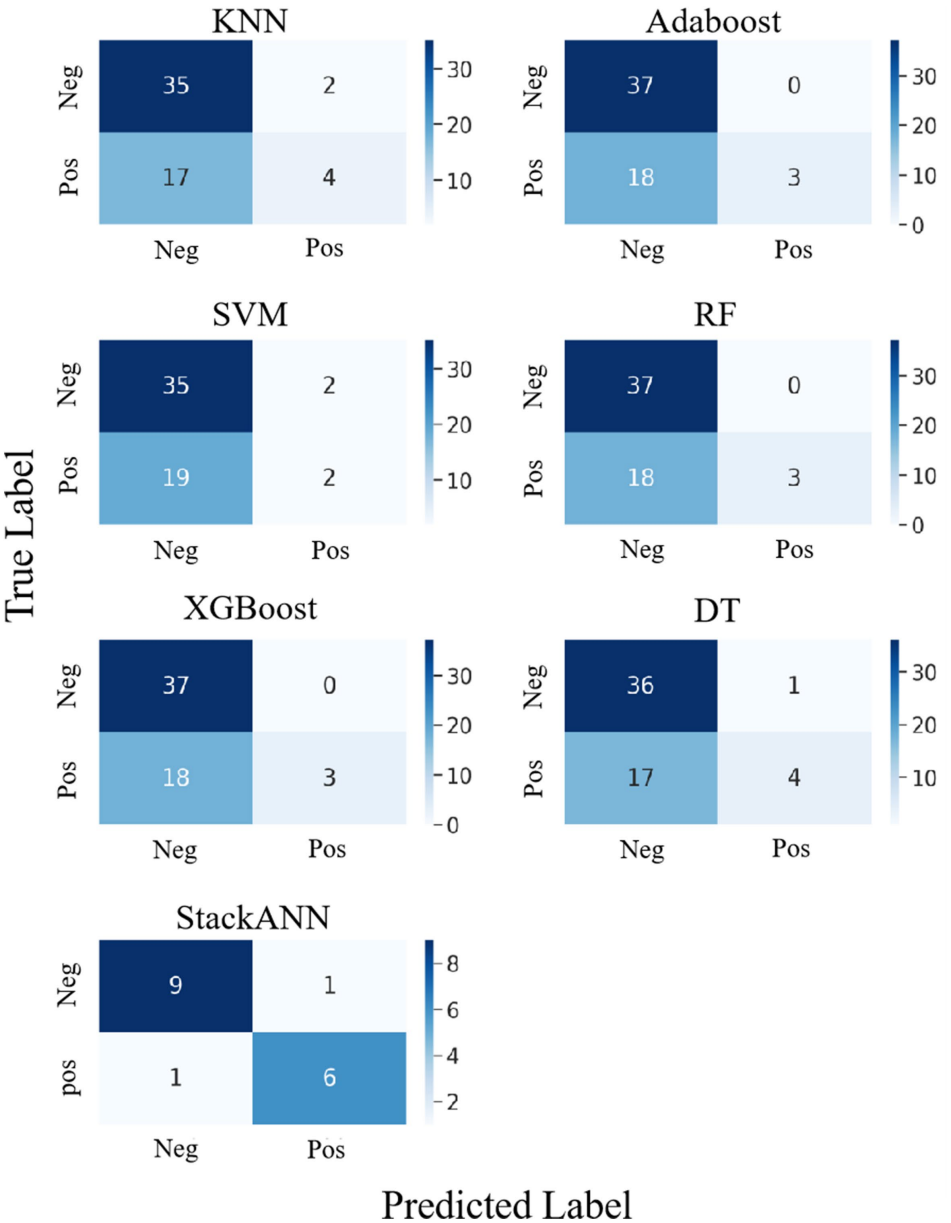
Confusion matrix comparison of StackANN model and baseline model on LBC dataset.

In the experiment of LBC dataset, StackANN was used as a stacking model to compare the classification results with those of the baseline models. From the results in Figure 6, among the six baseline models, XGBoost, AdaBoost and RF performed consistently in the classification results. Their confusion matrices showed that the models successfully identified 37 negative examples (TN = 37) and did not misjudge any negative examples as positive examples (FP = 0), indicating that these three models have high accuracy in the classification of benign samples. However, the performance in the identification of malignant samples was very weak, with only 3 positive examples correctly predicted (TP = 3) and 18 missed (FN = 18), showing a high risk of missed diagnosis. In contrast, KNN was slightly inferior in the classification of negative examples, with only 35 negative examples (TN = 35) identified and 2 false positives (FP = 2), but it was slightly improved in the classification of positive examples, with 4 positive examples correctly identified (TP = 4) and 17 missed (FN = 17), but the ability to identify malignant samples was still weak. SVM performs the same as KNN in negative example recognition (TN = 35, FP = 2), but is more insufficient in positive example recognition, with only 2 positive examples correctly classified (TP = 2) and 19 missed (FN = 19), making it the least sensitive to malignant samples among the six models. DT is slightly better than KNN and SVM in negative example recognition (TN = 36, FP = 1), and is on par with KNN in positive example recognition (TP = 4, FN = 17). In general, the six baseline models perform well in the recognition of benign tumors and have high classification ACC; however, there is a common problem of missed diagnosis in the recognition of malignant tumors. This will lead to the failure of key disease warnings and seriously affect clinical decision-making. In addition, the dataset of the baseline model has a sample imbalance problem, with more benign samples than malignant samples, which will affect the model’s tendency to learn the features of the majority class (negative examples), resulting in poor performance in the recognition of the minority class (positive examples), resulting in a high missed diagnosis rate.

Compared with the above baseline model, the optimized StackANN model showed obvious advantages in positive example recognition ability, identifying a total of 6 positive examples (TP = 7) and missing only 1 positive example (FN = 1), significantly reducing the missed detection rate of malignant tumors. At the same time, the negative example recognition performance was TN = 8 and FP = 1. Although the Sp decreased, the overall improvement in the positive example Recall rate was more clinically valuable. This result shows that the StackANN model can effectively alleviate the shortcomings of the traditional baseline model in positive example recognition while improving the model Recall, and has stronger practical application potential. In addition, the relative balance of samples (the number of positive samples is 8 and the number of negative samples is 9) helps to optimize the performance of the StackANN model, further supporting its advantage in positive example classification.

**Figure 7:**
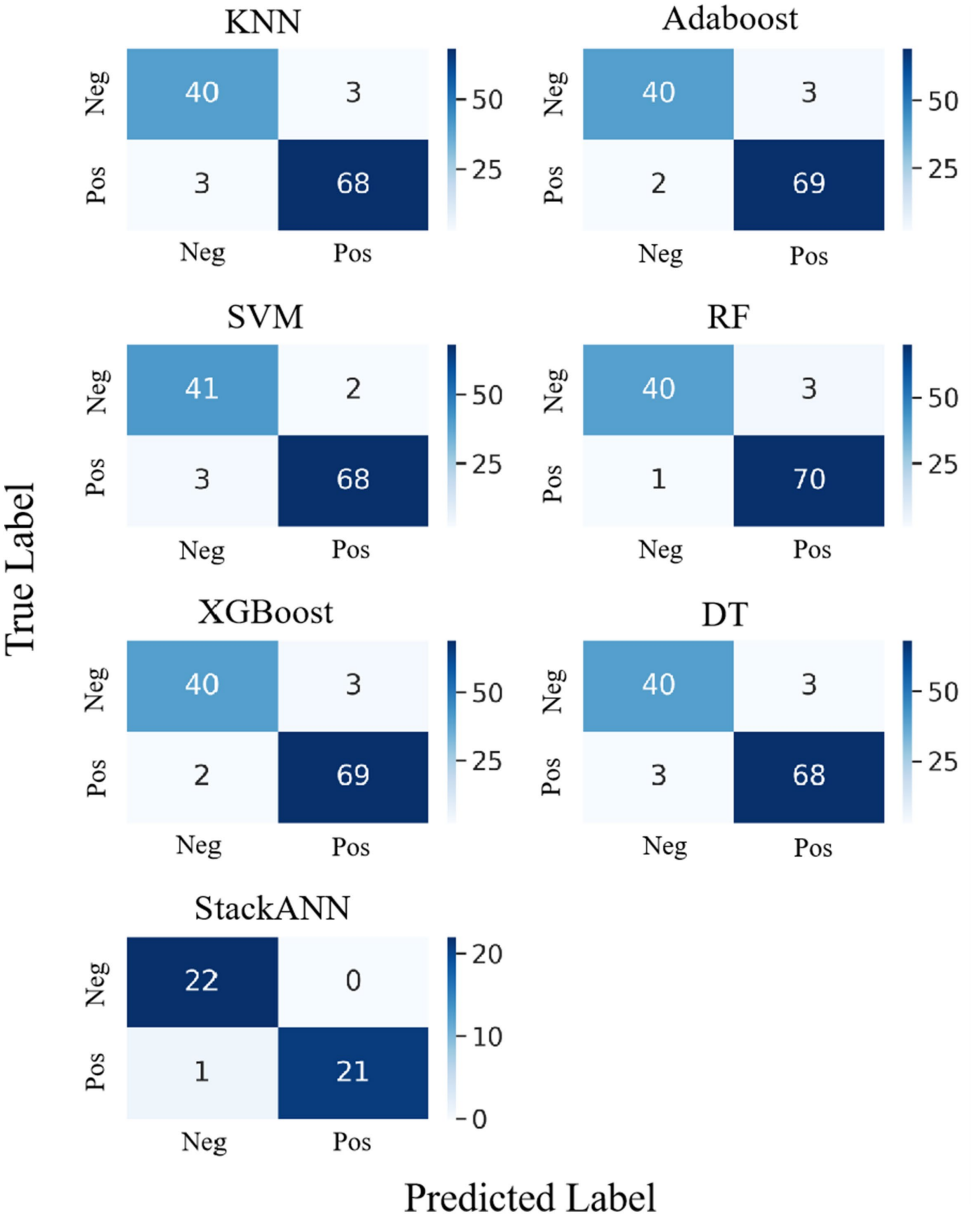
Confusion matrix comparison of StackANN model and baseline model on WDBC dataset.

The experimental results on the WDBC dataset are shown in Figure 7, which shows the confusion matrix comparison between the StackANN model and the six baseline models. Overall, the baseline models performed well in the identification of both positive and negative examples, with generally low numbers of FP and FN. Among them, KNN and DT had relatively high numbers of errors in both categories, both FP = 3 and FN = 3. However, in comparison, the StackANN model only missed one positive example (FN = 0) while keeping the false positive zero (FP = 1), showing better classification performance, especially in reducing missed diagnoses. In addition, the sample distribution of the optimized StackANN model is more balanced, with 32 positive samples and 33 negative samples, while the data used by the baseline model has 71 positive samples and 43 negative samples, which is imbalanced to a certain extent. In summary, the StackANN model shows higher classification ACC and lower misclassification rate when processing imbalanced datasets, especially in reducing missed diagnoses, proving its potential and effectiveness in practical applications.

### 3.4 External validation and cross-dataset generalization evaluation

**Figure 8:**
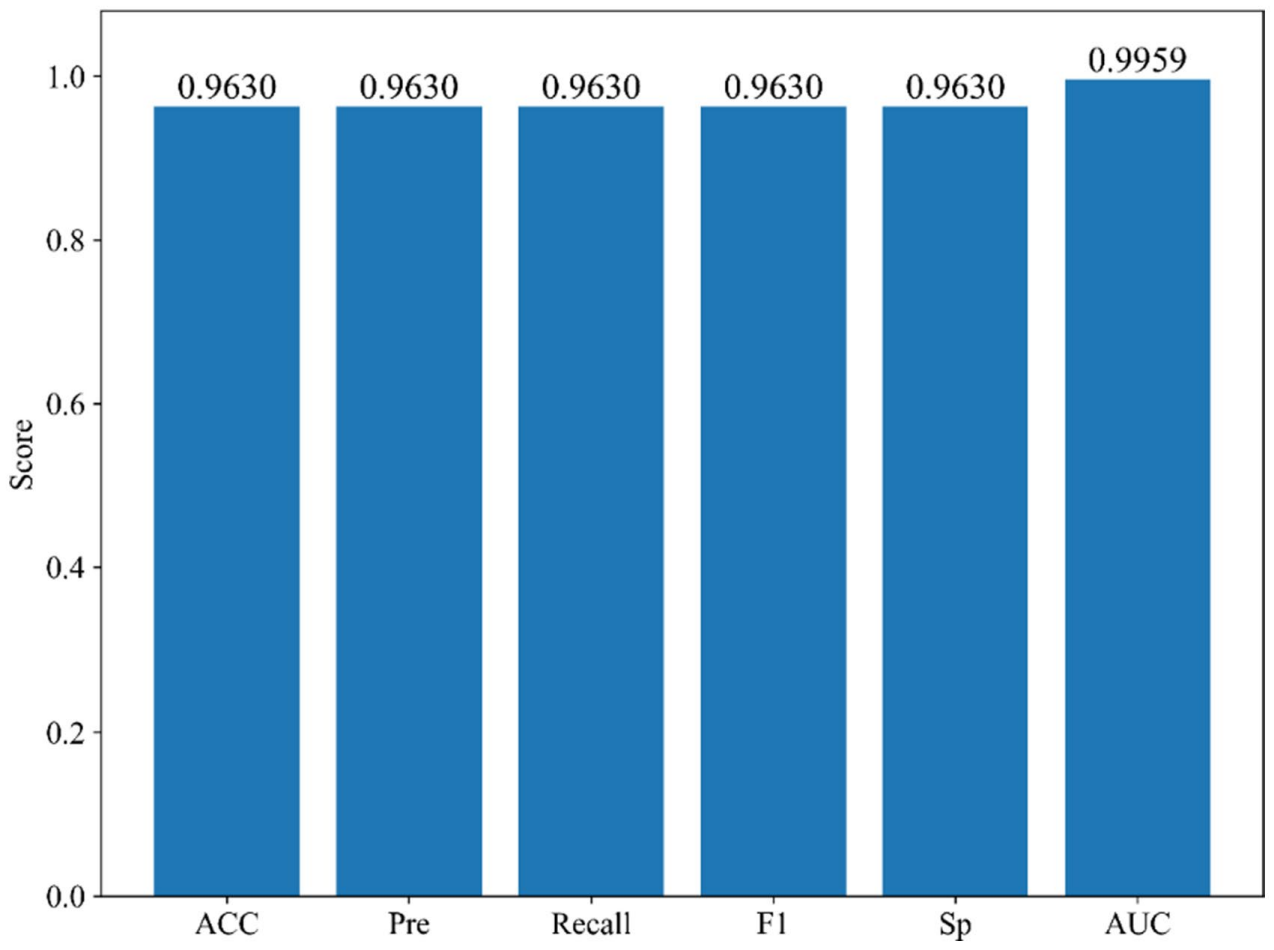
Confusion matrix comparison of StackANN model and baseline model on WDBC dataset.

To further assess the robustness and generalization of the proposed StackANN model in real-world clinical applications, we employed the WBCD as an independent external validation set and ensured comparability by strictly following the same preprocessing and normalization pipeline as applied to the WDBC dataset. As illustrated in Figure 8, StackANN delivered consistently strong performance across all key metrics on the WBCD dataset, achieving ACC, Pre, Recall, F1, and Sp values of 0.9630, with an outstanding AUC of 0.9959. The high consistency among these indicators highlights the model’s desirable balance between sensitivity and specificity, which is critical in minimizing both false positives and false negatives in medical diagnosis. Importantly, the exceptionally high AUC underscores StackANN’s strong discriminative capacity in distinguishing malignant from benign breast cancer cases, even under different feature spaces and sample distributions. These findings confirm that StackANN not only preserves superior diagnostic capability across multiple datasets but also exhibits resilience to variations in data characteristics, with results on the WBCD dataset remaining stable and consistent with those on the WDBC dataset. Clinically, this external validation underscores the practical applicability of StackANN, as its ability to generalize across datasets collected under diverse conditions and feature sets is essential for reliable deployment in multi-center and real-world hospital environments (47). Moreover, its stable performance indicates reduced risk of model degradation in new patient populations, which is a key prerequisite for safe clinical adoption. In conclusion, the external validation experiments demonstrate that StackANN achieves excellent generalization and stability, reinforcing its potential as a clinically valuable tool for breast cancer diagnosis and providing strong evidence to support its future large-scale, multi-institutional application.

### 3.5 Multiclassification assessment of breast Cancer subtypes

In breast cancer diagnosis, beyond the traditional binary classification of benign versus malignant, finer-grained classifications such as Basal-like, HER2-enriched, Luminal A, Luminal B, Normal-like, and Claudin-low subtypes hold significant clinical value and can guide personalized treatment (48). To extend the original StackANN model, which was designed for binary classification, to a multi-class setting, the following adjustments are required: first, use a LabelEncoder to encode each subtype label as an integer so that the model can handle multiple class outputs; second, each base model (KNN, AdaBoost, SVM, RF, XGBoost, DT) predicts the probability of each sample belonging to each class, and these probabilities are concatenated to form new feature vectors, which serve as inputs to the ANN meta-learner; finally, the ANN output layer is configured with a number of nodes equal to the number of classes, with each node corresponding to the predicted probability of a subtype, thereby enabling multi-class prediction.

We conducted experiments on the METABRIC2 dataset, which includes six breast cancer subtypes (49). This dataset was jointly constructed and provided by the Canadian Cancer Society Research Institute and its international collaborators. It contains comprehensive data from 1,980 patients with primary breast cancer, including gene expression data, clinical pathological features, and long-term survival information for each sample. For our breast cancer subtype classification study, we extracted gene expression profiles and clinical features, totaling 505 features. To meet the input requirements of machine learning algorithms, we performed digital encoding of categorical variables. For example, ER and PR statuses were mapped from “Positive/Negative” to numeric values of 1/0. In addition, we processed missing values to ensure the integrity and quality of the data. Based on the predictions of the StackANN model, we calculated multiple performance metrics for each subtype, including overall ACC, Pre, Recall, F1, Sp, and AUC. Here, ACC represents the overall correctness of the model across all samples; Pre, Recall, and F1 are calculated for each class, reflecting the model’s performance on individual subtypes; Sp and AUC are computed using a one-vs-rest (OvR) strategy to evaluate the model’s ability to distinguish a specific subtype from all others. The results of these metrics are summarized in the Table 5.

**Table 5:** Performance of the StackANN model in the METABRIC2 breast cancer multi-subtype classification task.

Subtype	ACC	Pre	Recall	F1	Sp	AUC
LumA	0.9266	0.8667	0.8966	0.8814	0.9730	0.9860
LumB	0.9266	0.9600	0.8000	0.8727	0.9932	0.9875
Her2	0.9266	0.9032	0.9655	0.9333	0.9797	0.9984
Basal	0.9266	0.9375	1.0000	0.9677	0.9864	1.0000
Normal	0.9266	0.9355	0.9667	0.9508	0.9864	0.9966
Claudin-low	0.9266	0.9643	0.9310	0.9474	0.9932	0.9991

The experimental results on the METABRIC2 dataset demonstrate that the StackANN model performs excellently in classifying six breast cancer subtypes. The overall ACC is consistently 0.9266 across all subtypes, indicating stable general classification capability. Specifically, the LumA subtype shows a Pre of 0.8667, Recall of 0.8966, and F1 of 0.8814, suggesting a good balance between Pre and Recall for LumA samples. LumB achieves a high Pre of 0.9600 but a relatively lower Recall of 0.8000, indicating that some LumB samples may be misclassified. Her2 and Basal subtypes have Recalls of 0.9655 and 1.0000, and F1 of 0.9333 and 0.9677, showing the model effectively identifies high-risk subtypes, especially Basal samples, which are almost perfectly captured. Normal and Claudin-low subtypes also demonstrate robust performance, with Pre of 0.9355 and 0.9643, F1 of 0.9508 and 0.9474, Sp above 0.98, and AUC close to 1, indicating strong capability in distinguishing these subtypes from others. Overall, StackANN exhibits high ACC, Recall, and Sp in multi-class breast cancer subtype classification, with particularly strong performance on critical high-risk subtypes (Basal and Claudin-low), highlighting its potential clinical utility for multi-subtype diagnosis.

### 3.6 Discussion on deployment and computing efficiency optimization

Although StackANN demonstrates excellent accuracy and robustness in breast cancer diagnosis, its relatively complex model structure may impose a computational burden in real-world hospital environments, particularly in primary healthcare settings or scenarios with limited computational resources. In our experiments, we verified that StackANN can perform inference on standard CPU environments, indicating that the model remains feasible under resource-constrained conditions. However, to further enhance efficiency and response speed in real-time clinical applications, multiple optimization strategies should be considered.

First, model pruning and quantization techniques can reduce the number of model parameters and storage requirements, thereby significantly shortening inference latency while maintaining performance close to the original model (50). Second, knowledge distillation can be employed to train a lightweight student model, achieving faster inference speed while preserving StackANN’s classification performance as much as possible (51). In addition, feature selection and dimensionality reduction methods (e.g., Principal Component Analysis (PCA), LASSO) can lower the input feature dimensions, reducing computational load and improving model interpretability, which provides clinicians with more intuitive decision support. Finally, deploying the model on optimized inference frameworks (e.g., TensorRT or ONNX Runtime), combined with hardware acceleration via GPU, FPGA, or other devices, can further reduce response time to meet real-time diagnostic requirements (52).

Future work should systematically evaluate these optimization strategies to balance StackANN’s diagnostic accuracy with real-time performance, ensuring that the model provides high-precision predictions while adapting to diverse hardware conditions and resource constraints in clinical applications.

## 4 Conclusion

This study proposes StackANN, a stacking ensemble framework that integrates multiple classical machine learning models with an ANN meta-learner, achieving superior performance in breast cancer classification. Experiments on the LBC, WDBC, and WBCD datasets demonstrated that StackANN consistently outperforms single models and recent hybrid approaches, particularly in identifying malignant cases with high Recall and balanced overall metrics. SHAP-based feature analysis further confirmed that the model’s decisions align with key clinical indicators such as tumor malignancy, size, and morphology. These results highlight StackANN’s robustness, generalization ability, and clinical relevance. While current validation remains limited, future work will focus on large-scale, multi-center external datasets and advanced techniques such as transfer learning to further enhance its clinical applicability. Overall, StackANN shows strong potential as a reliable, interpretable, and practical tool to support early breast cancer screening and diagnosis.

## Data Availability

The original contributions presented in the study are included in the article/Supplementary material, further inquiries can be directed to the corresponding author/s.
