# Supplementary material for: A robust stacked neural network approach for early and accurate breast cancer diagnosis
Source: Front Med (Lausanne). 2025 Oct 16;12:1644857. doi: 10.3389/fmed.2025.1644857 (PMC12571923; doi:10.3389/fmed.2025.1644857)
Supplement: Supplementary file 1 [file Table_1.docx]

**Table S1** Optimized Hyperparameters for Base Learners on LBC and WDBC Datasets

| Model | Hyperparameter | Optimized Value (LBC) | Optimized Value (WDBC) |
| --- | --- | --- | --- |
| KNN | n_neighbors | 20 | 5 |
| **AdaBoost** | n_estimators | 100 | 100 |
|  | learning_rate | 0.01 | 0.1 |
| **SVM** | C | 1 | 1 |
|  | kernel | 'rbf' | 'linear' |
|  | gamma | 'scale' | 'scale' |
| RF | n_estimators | 50 | 100 |
|  | max_depth | 10 | 10 |
|  | min_samples_leaf | 4 | 1 |
|  | min_samples_split | 2 | 2 |
| **XGBoost** | n_estimators | 150 | 100 |
|  | learning_rate | 0.01 | 0.3 |
|  | max_depth | 3 | 6 |
|  | subsample | 0.8 | 1 |
|  | colsample_bytree | 1 | 1 |
| **DT** | criterion | 'gini' | 'gini' |
|  | max_depth | 3 | 10 |
|  | min_samples_leaf | 1 | 1 |
|  | min_samples_split | 10 | 2 |

The data preprocessing process of the LBC Dataset：

First, we split the tumor_position column into three new columns (pos_1, pos_2, and pos_3) to more clearly describe the spatial location of the tumor. Then, for the three categorical variables of age, breast, and tumor_size, we applied the label encoding technique to convert these categorical variables into numerical variables to meet the input requirements of the machine learning algorithm. Specifically, different age ranges in the age column are mapped to integer values: '20-30' is mapped to 0, '30-40' is mapped to 1, '40-50' is mapped to 2, '50-60' is mapped to 3, and '60+' is mapped to 4. For the breast column, which represents the side of the breast lump, 'left' is mapped to 0 and 'right' is mapped to 1. The tumor size categories in the tumor_size column are encoded in order from small to large, where 'very small' is 0, 'small' is 1, 'medium' is 2, 'big' is 3, 'very big' is 4, and 'urgent treatment' is 5. These data set processing ensures that the category information in the original data is reasonably converted, laying a solid foundation for the subsequent machine learning modeling process.
